# Performance of Trifocal Intraocular Lens Implantation in Highly Myopic Patients With and Without Astigmatism

**DOI:** 10.7759/cureus.89755

**Published:** 2025-08-10

**Authors:** Curtis R Martin, Ayorinde Cooley, Fatma Shakarchi, Brittany B DeNaro, Timothy Link, Nicholas Zaunbrecher, Christopher Shelby, Wyche T Coleman, Stephen LoBue

**Affiliations:** 1 Department of Ophthalmology, Willis-Knighton Medical Center, Shreveport, USA

**Keywords:** astigmatism treatment, cataract treatment, high myopia, trifocal iols, visual acuity outcome

## Abstract

Purpose: To analyze the performance of trifocal intraocular lens (IOL) implantation in patients with high myopia vs patients with low-to-moderate myopia, with or without astigmatism.

Methods: A retrospective study examining trifocal IOL implantation in myopic patients was conducted at a single institution by one surgeon. Patients were followed on postoperative day 1, at one month, and at three months. Refraction was performed between the first and third months. A previously validated 10-question survey, scored from 0-10, was conducted one month after surgery. Patients were grouped into high myopia (≤ −5.00 diopters (D), axial length >25 mm) and low-to-moderate myopia (−1.00 to −5.00 D, axial length <25 mm). Exclusion criteria included high spherical aberration above 0.6, macular pathology, glaucoma, corneal disease, or scarring. Refractive outcomes, uncorrected distance visual acuity (UDVA), uncorrected near visual acuity (UNVA), and patient satisfaction surveys were compared.

Results: A total of 72 eyes were included in the study (26 high myopes and 46 low-to-moderate myopes). The axial length and sphere were 26.67 ± 1.44 mm and -7.29 ± 2.80 D in the high myopia group vs 24.10 ± 1.11 mm and -2.38 ± 1.86 D in the low-to-moderate myopia group, p<0.001 and p<0.001, respectively. At one month postoperatively, UDVA, UNVA, and spherical equivalent (SE) revealed no difference between myopic groups. Residual refractive errors were ≤0.5 D in >90% of high (n=46) and low-to-moderate myopes (n=26). Comparing patients with >1.0 D of astigmatism in both high myopic (n=14) and low-to-moderate myopic groups (n=24) revealed no significant difference in sphere, cylinder, SE, monocular and binocular UDVA or UNVA, p>0.05. Overall patient satisfaction and the presence and severity of dysphotopsias were also similar between all groups, p>0.05.

Conclusion: Trifocal IOL may demonstrate high levels of visual performance and patient satisfaction in the presence of high axial myopia vs low-to-moderate myopia in patients with greater or less than 1.0 D of astigmatism in specific patients.

## Introduction

Myopia is the second most common cause of blindness and refractive visual impairment worldwide [1]. The estimated prevalence of myopia in 2000 ranged from 1.4 billion people, approximately 22.9% of the world's population, which is predicted to increase to 4.76 billion or 50% of the world's population by 2050 [2]. Furthermore, approximately 938 million people will be classified as having high myopia with a refractive error of at least -5 diopters (D) [2].

High myopia is associated with a greater risk of irreversible vision loss from glaucoma, retinal detachment, myopic macular degeneration, and cataracts [3]. With regard to cataract formation, high myopia patients have 3 and almost 8 times the risk of developing nuclear cataract formation and posterior subcapsular cataract, respectively [4]. Higher severity of myopia is associated with an increased incidence of cataract surgery [4].

High myopia may also be associated with increased intraocular difficulty due to the increased or fluctuating depth of the anterior chamber, large or floppy capsular bag, zonular weakness, and higher rates of retinal detachment. However, patients with myopia are often more motivated to become spectacle or contact lens independent after lens replacement. However, the performance of multifocal intraocular lens (MfIOL) remains controversial for patients with high myopia. Retinal pathology, lens decentration, or toric intraocular lens (IOL) rotation may limit optimal visual potential, inhibiting the performance of a MfIOL [5]. Few studies have compared the performance of MfIOL in patients with varying degrees of myopia. We have previously documented good outcomes with MfIOL even in cases of extreme myopia [6]. However, our results were limited to one case.

Thus, in this study, we expanded to a larger cohort of patients with or without astigmatism. Our aim is to compare the performance of trifocal IOL in various levels of myopia to determine refractive predictability, visual outcomes, patient satisfaction, and spectacle independence.

## Materials and methods

Study design

A retrospective study was conducted at the Willis-Knighton Eye Institute in Shreveport, Louisiana. The study was approved by the Institutional Review Board at Willis-Knighton Medical Center and was conducted according to the tenets of the Declaration of Helsinki. All patients provided written and verbal informed consent after their briefing on the study protocol.

Patients

Patients included in the study were men and women over 18 years of age who underwent cataract or clear lens extraction with posterior chamber IOL implantation between July 2022 and July 2023. Inclusion criteria involved low-to-moderate myopia defined as -1 to -5 D and an axial length <25 mm. High myopia was defined as -5 D or worse and an axial length >25 mm. Exclusion criteria included patients with a high spherical aberration above 0.6, macular pathology, glaucoma, corneal disease, or scarring.

IOL description

The AcrySof PanOptix Trifocal IOL (Alcon, Fort Worth, TX, USA) model TFAT00 vs TFAT30-60 was the primary lens used in the study. This trifocal IOL has a biconvex optic containing an aspheric design and 26 diffractive rings on the anterior surface. Multiple concentric rings on the IOL allow for a range of vision, including distance, intermediate, and near.

Procedures and assessments

Femtosecond laser-assisted cataract surgery (FLACS) was performed using the LenSx platform (Alcon, Fort Worth, TX, USA) to create a 5 mm anterior capsulotomy and fragmentation of the nucleus. The main incision and paracentesis were created manually. All phacoemulsification was performed with the Centurion Vision System (Alcon, Fort Worth, TX, USA) using topical anesthesia. Optiwave Refractive Analysis (ORA, Alcon, Fort Worth, TX, USA) was used after cataract extraction to verify or modify preoperative IOL selection. A soft polymer irrigation/aspiration tip was used to polish the posterior capsule. Cataract extraction was uncomplicated and completed in all patients with the placement of a trifocal TFAT00 vs TFAT30-60 IOL.

Visual acuity was measured using the Snellen chart, and the total number of letters read was recorded and converted to logMAR for analyses. Distance measurements for binocular and monocular uncorrected distance visual acuity (UDVA) were tested at 6.1 m under 100% contrast photopic conditions with ambient room lighting. All binocular and monocular uncorrected near visual acuity (UNVA) were tested between 40 cm and 33 cm at the patient’s preferred reading distance using a Rosenbaum near chart under photopic conditions with ambient room lighting.

Patients were followed on postoperative day 1, at one month, and at three months. The final refraction was performed between the first and third months. Manifest refractions were performed using the maximum plus refraction technique with a plus cylinder. The Refractive Cataract Surgery Survey (RCSS), a validated, 10-question survey to measure postoperative performance and patient satisfaction, was conducted one month after surgery.

The survey comprised 10 questions. The first seven questions were answered on a 1-10 scale where patients were asked to rate their satisfaction with different aspects of vision and overall lens choice. The remaining three were binary (yes/no) questions that assessed postoperative outcomes such as glare and halo perception and their impact on daily activities. 

Endpoint

The study endpoints included the assessment of monocular and binocular UDVA, UNVA, and subjective vision quality, which were evaluated with a questionnaire between low-to-moderate myopia and high myopia groups, with and without astigmatism.

Statistical analysis

IBM SPSS Statistics for Windows, Version 30.0 (Released 2024; IBM Corp., Armonk, New York, USA) was used to make statistical comparisons for each group. The data groups are presented as mean ± standard deviation when possible. Independent t-tests for numeric variables and chi-square tests for categorical variables were utilized for statistical comparisons. The significance level was set at p<0.05. 

## Results

A total of 72 eyes from 36 patients were included in the study from July 2022 to July 2023. The high myopia group included 26 eyes from 13 patients, while the low-to-moderate myopia group included 46 eyes from 23 patients (Table 1). The high myopia group had an axial length of 26.7 ± 1.4 mm and a spherical equivalent of -7.3 ± 2.8 D compared to the low-to-moderate myopia group with 24.1 ± 1.1 mm and -2.4 ± 1.9 D, p<0.001 and p<0.001, respectively (Table 1).

**Table 1:** Preoperative measurements for all patients in the cohort. D, diopters; K, keratometry value; IOL, intraocular lens.

	Axial length (mm)	K1 (D)	K2 (D)	IOL (D)	Sphere (D)	Cylinder (D)	Axis (degrees)
High myopia (n=26)	26.7 ± 1.4	43.6 ± 1.3	43.8 ± 3.2	11.9 ± 3.1	-7.3 ± 2.8	0.45 ± 1.0	86 ± 68
Low/moderate myopia (n=46)	24.1 ± 1.1	43.5 ± 2.0	44.5 ± 2.1	19.1 ± 3.1	-2.38 ± 1.9	0.85 ± 1.1	103 ± 63
p-value	p<0.001	p>0.05	p>0.05	p<0.001	p<0.001	p>0.05	p>0.05

A total of 38 eyes from 19 patients were corrected for astigmatism. The high myopia group involved 14 eyes with a mean preoperative cylinder of 1.5 ± 0.93, while the low-to-moderate group included 24 eyes with a mean preoperative cylinder of 1.44 ± 0.93.

Objective outcomes

**Figure 1:**
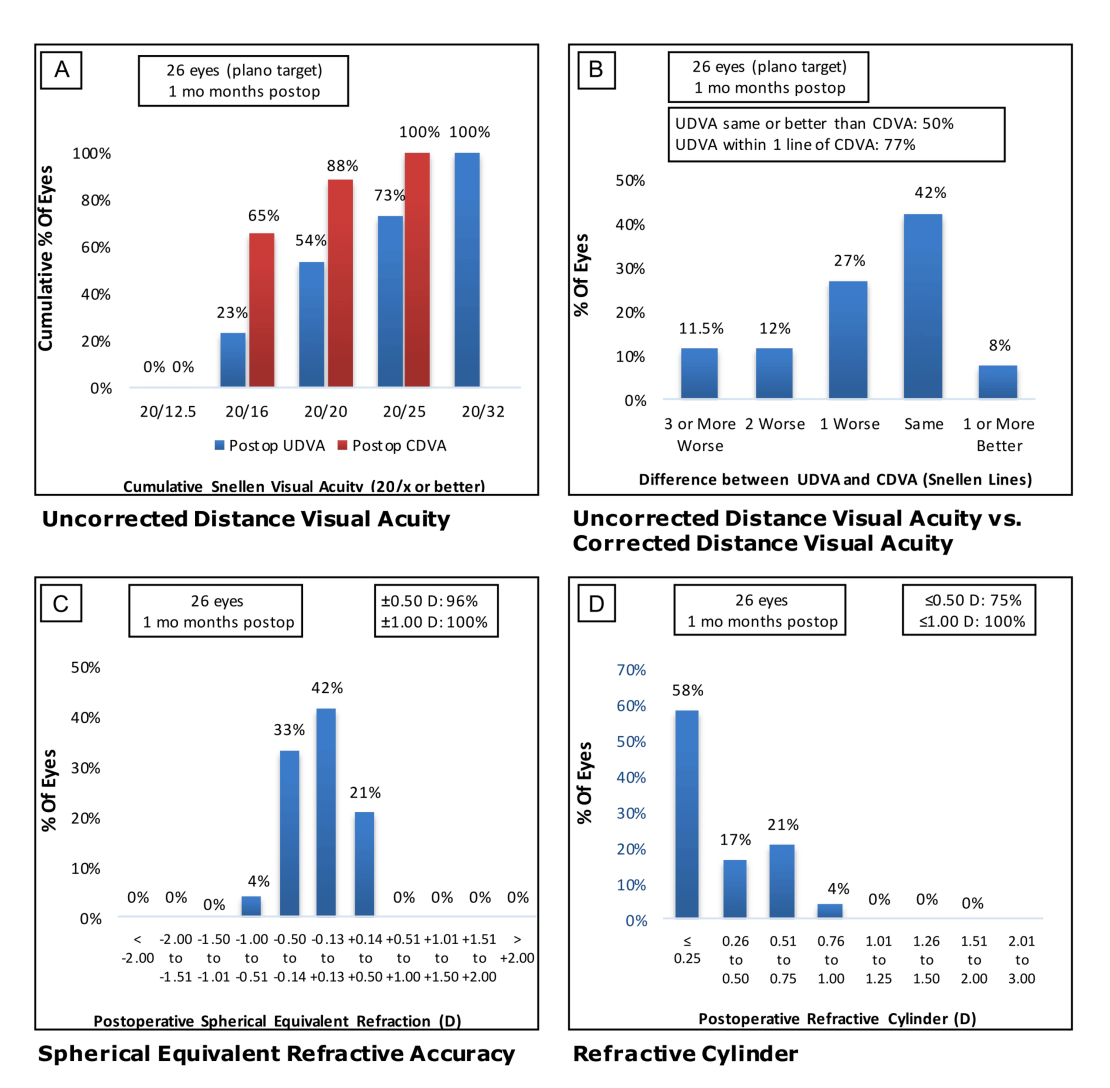
Visual acuities one month after implantation of trifocal IOL in the high myopia group. (A) Cumulative monocular UDVA (blue) and CDVA (red). (B) Comparison of UDVA and CDVA by difference in Snellen line readings. (C) Spherical equivalent refractive accuracy. (D) Residual astigmatism. IOL, intraocular lens; UDVA, uncorrected distance visual acuity; CDVA, corrected distance visual acuity.

The high myopia group had overall good monocular UDVA with 73% (n=19 eyes) of the cohort demonstrating 20/25 or greater visual acuity (Figure 1A). Monocular UDVA was the same or better than corrected distance visual acuity (CDVA) in 50% (n=13) of eyes or within one line in 77% (n=20) of eyes (Figure 1B). The high myopia group had low residual spherical and cylinder refractive errors with 96% (n=25) and 75% (n=20) of eyes within 0.5 D, respectively (Figure 1C and D).

**Figure 2:**
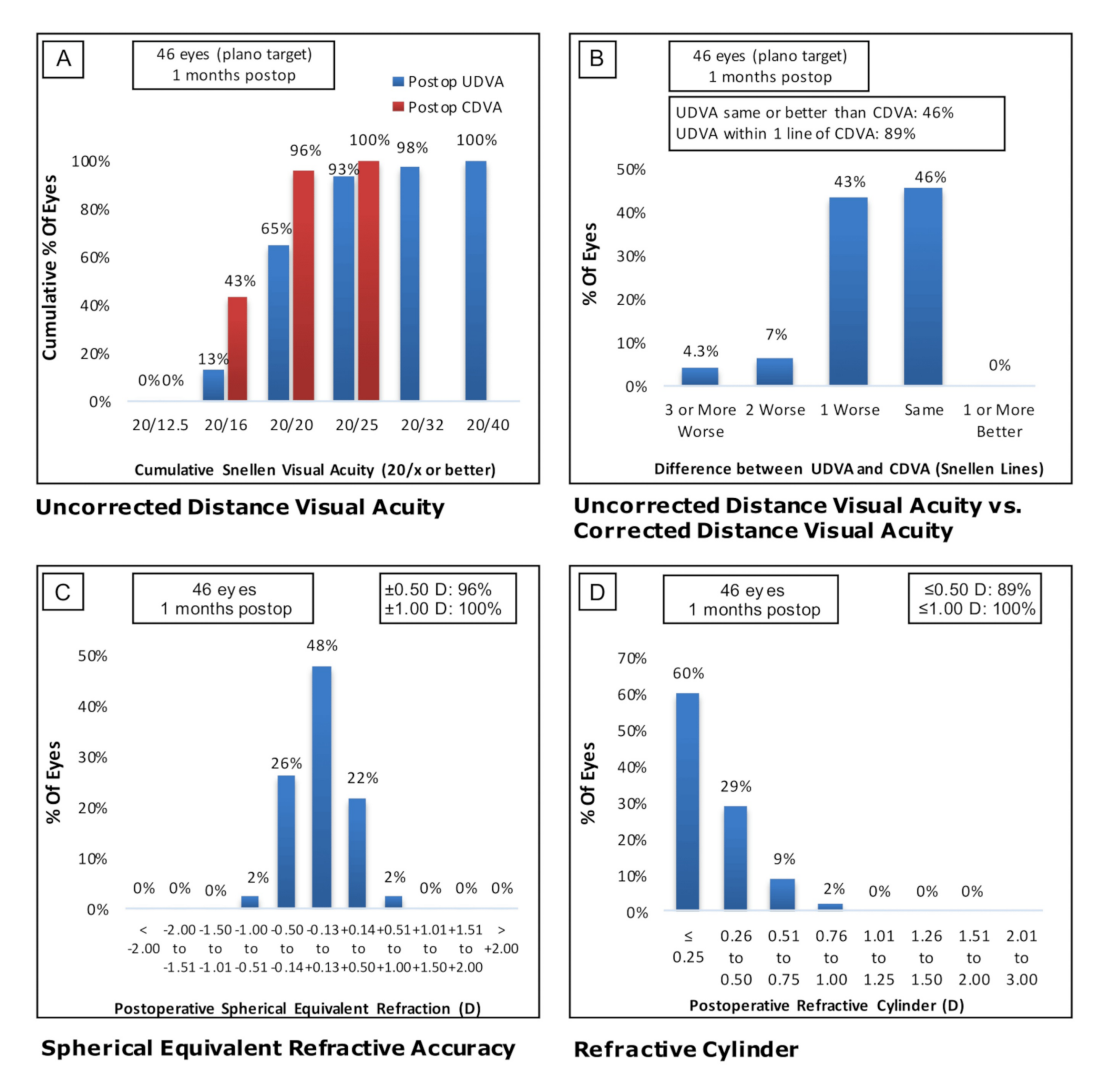
Visual acuities one month after implanting trifocal IOL in the low/moderate myopia group. (A) Cumulative monocular UDVA (blue) and CDVA (red). (B) Comparison of UDVA and CDVA by difference in Snellen line readings. (C) Spherical equivalent refractive accuracy. (D) Residual astigmatism. IOL: intraocular lens; UDVA, uncorrected distance visual acuity; CDVA, corrected distance visual acuity.

The low-to-moderate myopia group also had a good overall monocular UDVA with 93% (n=43 eyes) of the cohort demonstrating 20/25 or greater visual acuity (Figure 2A). The monocular UDVA was the same or better than CDVA in 46% (n=21) of eyes or within one line in 89% (n=41) of eyes (Figure 2B). The low-to-moderate myopia group had low residual spherical and cylinder refractive errors with 96% (n=44) and 89% (n=31) of eyes within 0.5 D, respectively (Figure 2C and D).

Analyzing both the high myopia and low-to-moderate myopia groups revealed no significant difference in postoperative sphere, cylinder, spherical equivalent (SE), and monocular UDVA and UNVA, with p>0.05 (Table 2). Binocular UDVA was significantly better in the high myopia group due to more challenging refractive patients involving two patients with 8-cut radial keratotomy (RK) and hyperopic laser-assisted in situ keratomileusis (LASIK) in the low-to-moderate myopia group. Excluding these two patients resulted in similar binocular uncorrected distance visual acuity (BUDVA) between both groups. Comparing patients with astigmatism in both myopic groups revealed no significant differences in sphere, cylinder, SE, and monocular and binocular UDVA or UNVA, with p>0.05 (Table 3).

**Table 2:** Postoperative visual acuity for all patients in the cohort. SE, spherical equivalent; MUDVA, monocular uncorrected distance visual acuity; MNVA, monocular near visual acuity; BUDVA, binocular uncorrected distance visual acuity; BNVA, binocular near visual acuity.

	Sphere	Cylinder	SE	MUDVA	MNVA	BUDVA	BNVA
High myopia (n=26)	-0.28 ± 0.3	0.39 ± 0.31	-0.09 ± 0.27	0.02 ± 0.11	0.08 ± 0.09	-0.05 ± 0.07	0.01 ± 0.03
Low/moderate myopia (n=46)	-0.20 ± 0.3	0.33 ± 0.24	-0.03 ± 0.27	0.05 ± 0.08	0.04 ± 0.06	0.00 ± 0.05	0.01 ± 0.03
p-value	p>0.05	p>0.05	p>0.05	p>0.05	p>0.05	p<0.05	p>0.05

**Table 3:** Postoperative visual acuity for patients with astigmatism. SE, spherical equivalent; MUDVA, monocular uncorrected distance visual acuity; MNVA, monocular near visual acuity; BUDVA, binocular uncorrected distance visual acuity; BNVA, binocular near visual acuity.

	Sphere	Cylinder	SE	MUDVA	MNVA	BUDVA	BNVA
High myopia (n=14)	-0.11 ± 0.25	0.36 ± 0.32	0.07 ± 0.24	0.06 ± 0.13	0.09 ± 0.06	-0.05 ± 0.09	0.00 ± 0.00
Low/moderate myopia (n=24)	-0.19 ± 0.33	0.36 ± 0.26	-0.01 ± 0.32	-0.03 ± 0.30	0.05 ± 0.06	0.00 ± 0.06	0.01 ± 0.03
p-value	p>0.05	p>0.05	p>0.05	p>0.05	p>0.05	p>0.05	p>0.05

**Figure 3:**
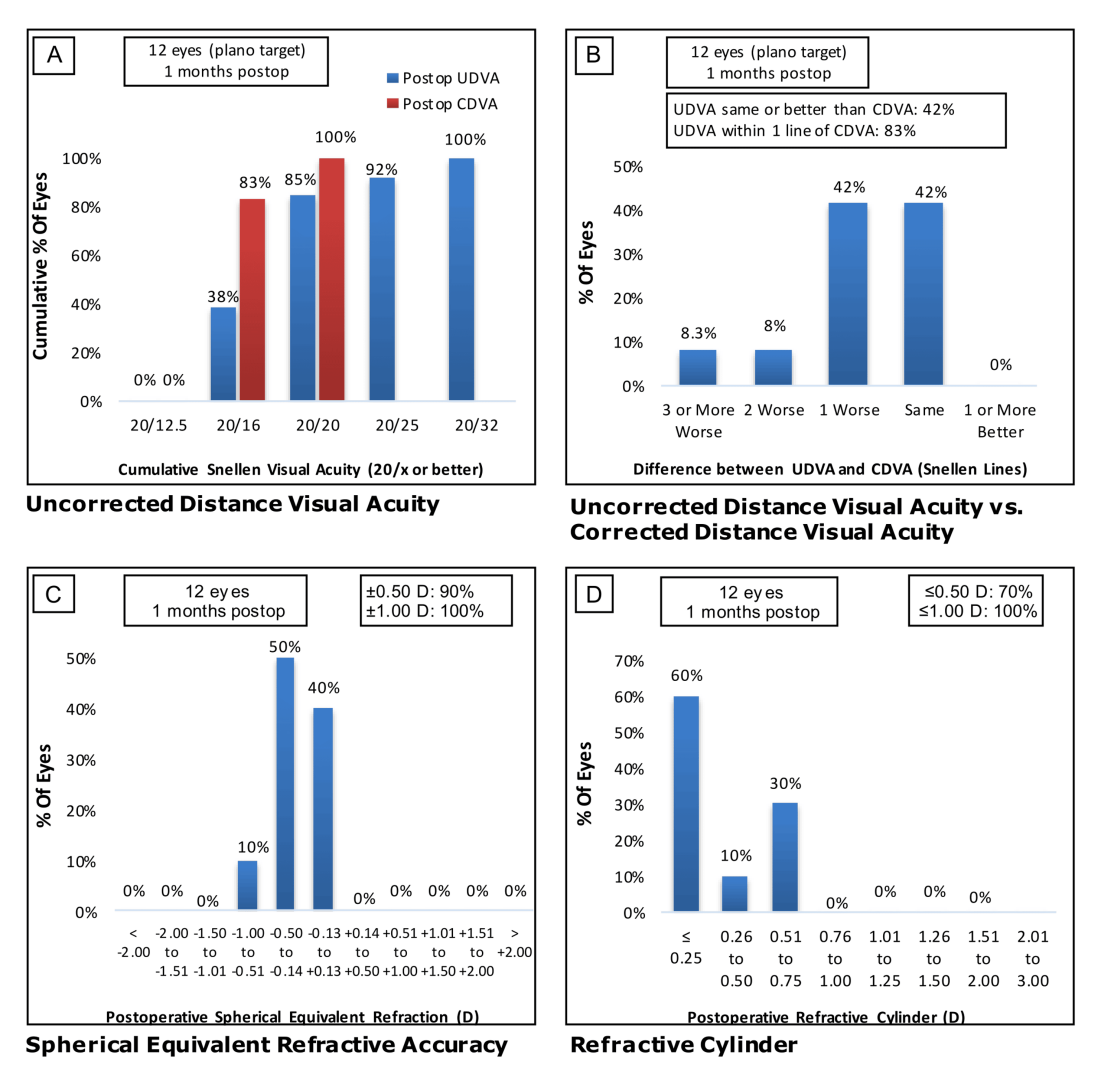
Visual acuities one month after implantation of trifocal IOL in the high myopia group without astigmatism. (A) Cumulative monocular UDVA (blue) and CDVA (red). (B) Comparison of UDVA and CDVA by difference in Snellen line readings. (C) Spherical equivalent refractive accuracy. (D) Residual astigmatism. IOL: intraocular lens; UDVA, uncorrected distance visual acuity; CDVA, corrected distance visual acuity.

**Figure 4:**
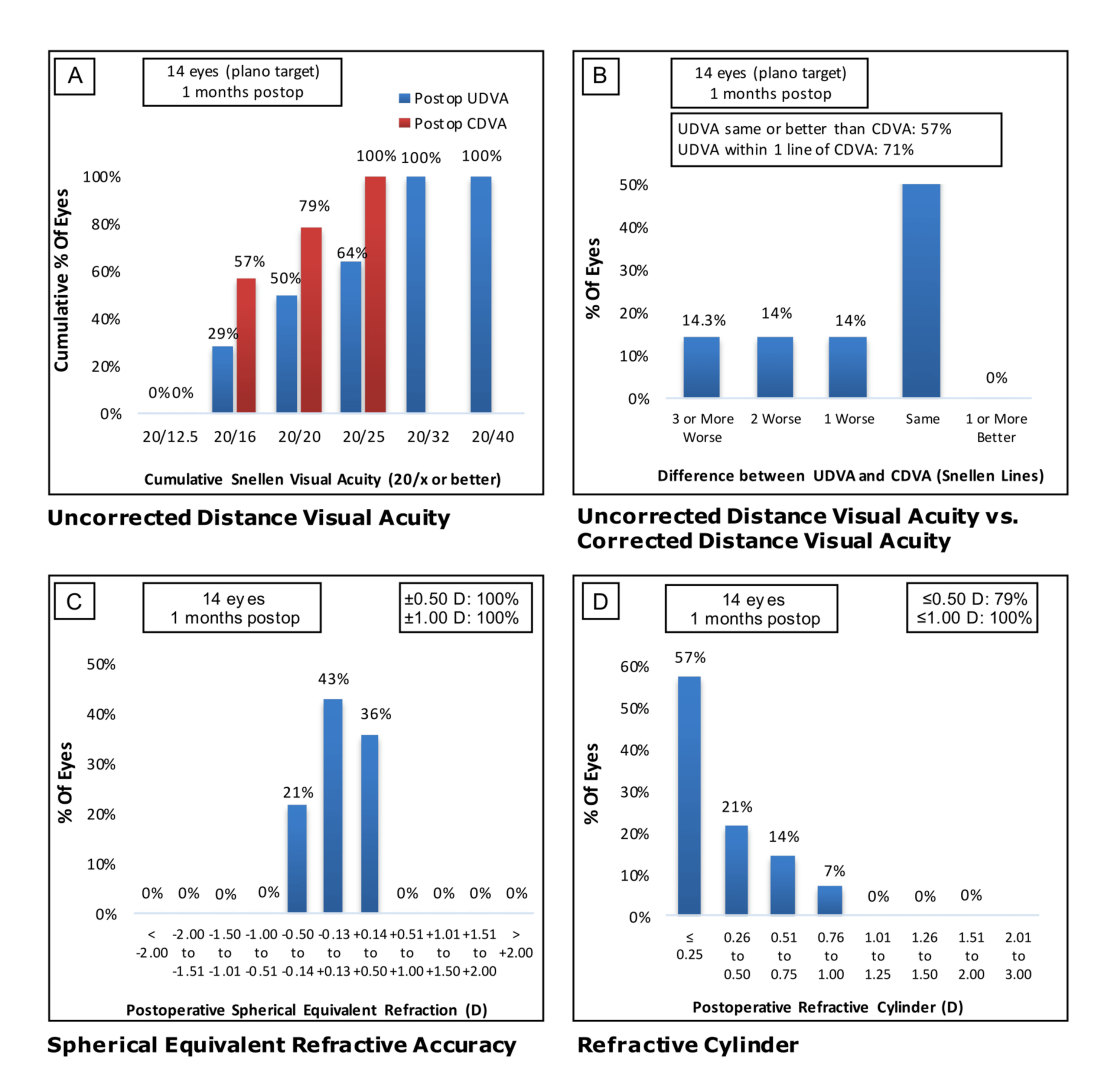
Visual acuities one month after implanting trifocal IOL in the high myopia group with astigmatism. (A) Cumulative monocular UDVA (blue) and CDVA (red). (B) Comparison of UDVA and CDVA by difference in Snellen line readings. (C) Spherical equivalent refractive accuracy. (D) Residual astigmatism. IOL, intraocular lens; UDVA, uncorrected distance visual acuity; CDVA, corrected distance visual acuity.

We further compared the high myopia group to patients with and without astigmatism. Without astigmatism, a total of 92% (n=11) of eyes had 20/25 or better monocular UDVA, with 83% (n=10) of eyes within one line of best CDVA (Figure 3A and B). The low astigmatism group had low residual spherical and cylinder refractive errors, with 90% (n=10) and 70% (n=8) of eyes within 0.5 D, respectively (Figure 3C and D). The high astigmatism group had 64% (n=9 eyes) with 20/25 or better UDVA, with 71% (n=10 eyes) within one line of CDVA (Figure 4A and B). The high astigmatism group had low residual spherical and cylinder refractive errors, with 100% (n=14)and 79% (n=11) of eyes within 0.5 D, respectively (Figure 4C and D).

Subjective outcomes

The visual performance questionnaires with questions 1-10 were performed one month after cataract surgery. All enrolled patients completed the survey (n=72). The visual performance was analyzed for the total cohort vs patients with significant preoperative astigmatism.

**Figure 5:**
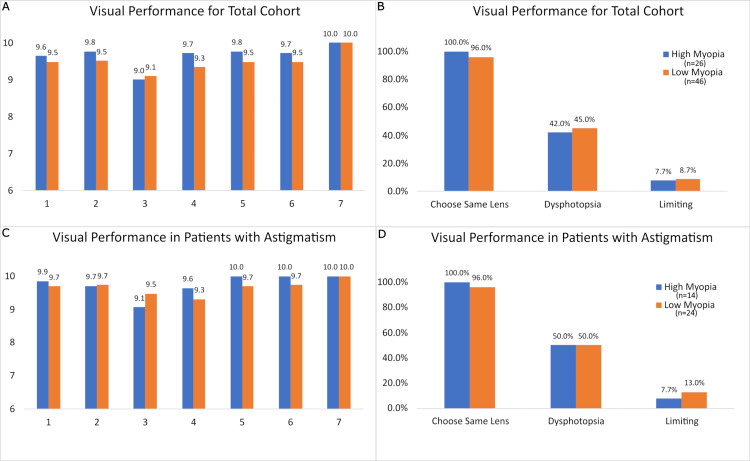
Refractive cataract surgery survey results. (A) Survey responses from the total cohort for questions 1-7, which assessed quality of distance (1), intermediate (2), and near (3) vision, glasses independence (4), met expectations (5), satisfaction with lens choice (6), and satisfaction with physician service (7) (n=72). (B) Survey responses from the total cohort to questions 8-10, which assessed if the patient would choose the same lens again and burden of dysphotopsias. Question 8: *X^2^*(1, n=72) = 1.65, p>0.05. Question 9: *X*^2^(1, n=72) = 0.08, p>0.05. Question 10: *X*^2^(1, n=72) 0.13, p>0.05. (C) Survey responses from patients with astigmatism for questions 1-7 (n=38). (D) Survey responses from patients with astigmatism for questions 8-10. Question 8: *X*^2^(1, n=38) = 0.12, p>0.05. Question 9: *X*^2^(1, n=38) = 0.01, p>0.05. Question 10: *X*^2^(1, n=9) = 0.03, p>0.05.

Questions 1-3 involving the quality of distance, intermediate, and near vision without glasses were similar in both groups, p>0.05 (Figure 5A). The level of glasses independence (question 4) and quality of vision meeting expectations (question 5A) were similar in both the total cohort and patients with significant preoperative astigmatism, p>0.05 (Figure 5A). The overall satisfaction with the lens and physician (questions 6 and 7) were similar in both the total cohort and patients with significant preoperative astigmatism, p>0.05 (Figure 5A).

Patients stating that they would choose the same lens again was documented in 100% (n=13) of patients with high myopia vs 96% (n=23) with low-to-moderate myopia within the total cohort, *X*^2^(1, n=72) = 1.65, p>0.05 (Figure 5B). The rate of dysphotopsias (e.g., glare) for the total cohort was 42% vs 45%, *X*^2^(1, n=72) = 0.08, p>0.05, for the high myopia and low-to-moderate myopia group, respectively (Figure 5B). The percentage of activity-limiting dysphotopsias for the total cohort was similar between the high myopia group (7.7%) and the low-to-moderate myopia group (8.7%), *X*^2^(1, n=33) = 0.13, p>0.05 (Figure 5B).

For patients with high or low-to-moderate myopia with significant astigmatism, responses to the RCSS were similar between both groups for questions 1-7, p>0.05 (Figure 5C). The proportion of patients who stated they would choose the same lens again was 100% (n=7) in the high myopia group and 96% (n=12) in the low-to-moderate myopia group within the astigmatism subset, *X*^2^(1, n=38) = 0.12, p>0.05 (Figure 5D). The rate of dysphotopsias for eyes with significant astigmatism was 50% in both groups, *X*^2^(1, n=38) = 0.01, p>0.05 (Figure 5D). The percentage of limiting dysphotopsias for eyes with significant astigmatism was 7.7% and 13% for high myopia and low-to-moderate myopia groups, respectively, *X*^2^(1, n=9) = 0.03, p>0.05 (Figure 5D).

## Discussion

Trifocal IOL are designed to provide clear vision at multiple distances. Highly myopic individuals are typically glasses or contact lens-dependent after cataract surgery. Trifocal IOL are proposed to provide spectacle independence and higher patient satisfaction with their postoperative vision compared to conventional monofocal lenses [7]. The prevalence of high myopia is expected to increase significantly worldwide by 2050 [2]. Therefore, developing and evaluating emerging therapeutic strategies to optimize patient visual outcomes is necessary.

This study aimed to analyze and assess visual and refractive outcomes after the implantation of trifocal IOL in patients with high myopia compared to patients with low myopia. Previous studies have reported that PanOptix IOL implantation provides excellent visual acuity across short, intermediate, and long distances in eyes with high myopia [8]. Studies on the long-term clinical outcomes of this IOL have shown stable clinical outcomes for visual acuity and refractive error [9]. Trifocal IOL can maintain good functional vision (greater than 0.30 logMAR) in highly myopic eyes at near, intermediate, and distance ranges [10-12]. A systematic review by Zhang et al. demonstrated better uncorrected near and intermediate visual acuity in patients with trifocal IOL compared to bifocal IOL and that trifocal IOL were associated with a reduced incidence of posterior capsular opacification formation [13].

In our study of 36 patients and 72 eyes, the high myopia group showed good visual outcomes one month postop, with 100% (n=26 eyes) of patients having binocular UDVA of 20/32 or better (Figure 1A) and 50% (n=13 eyes) of patients with UDVA being the same or better than CDVA (Figure 1B). These results are comparable to those of other studies that reported UDVAs of 20/32 or better in 92.3 to 100% of patients in the early postoperative period after trifocal IOL implantation [14,15]. Postoperative spherical and cylinder refractive errors were within 0.50 D for 96% (n=25) and 75% (n=20) of eyes, respectively (Figure 1C and D). No significant differences in postoperative UDVA or refractive errors were found between the high and low-to-moderate myopia groups (Figure 2A-D), suggesting similar visual outcomes occur despite the initial degree of myopia. These findings are consistent with other studies, which note that significant differences in visual outcomes between low and high myopes become more likely in cases of extremely high myopia (axial length >28 mm) [11,16]. We found there was no significant difference in postoperative UNVA within our patient cohort, suggesting trifocal IOL are effective for providing good near vision across varying degrees of myopia regardless of astigmatism. We observed a significant difference in BUDVA in the high myopia group compared to the low/moderate group when including patients with previous refractive treatments (RK and LASIK). However, after excluding corneal-refractive patients, there was no difference in UDVA between groups. Interestingly, past studies have shown that a hybrid diffractive-refractive approach can produce excellent visual and refractive outcomes, and the use of trifocal IOL with this approach leads to significantly better UNVA [17,18].

Residual refractive errors reduce the benefits of IOL placement and thus prevent patients from achieving spectacle independence [19,20]. In trifocal IOL, residual astigmatism can alter focus at varying distances [21], affecting objective and subjective visual quality [22]. Several groups have reported that limiting residual astigmatism to ideally 0.50 D with an upper limit of 0.75 D is optimal for preventing functional deficiencies in vision after IOL implantation [22,23]. We compared outcomes for patients with low (Figure 3A-D) and high (Figure 4A-D) preoperative astigmatism within the high myopia group. Low residual spherical and cylinder refractive errors were largely achieved in both groups. In the high astigmatism group, 7% (n=1) of eyes had a postoperative refractive cylinder of 0.76 to 1.00 D, but with no statistically significant difference in residual astigmatism between the two groups. These outcomes may reflect the difficulty of treating astigmatism with IOL in high myopia due to factors such as more stringent IOL power calculations and increased risk of IOL rotation in combination with the high preoperative astigmatism in these patients. Our findings are consistent with Orts-Vila et al., who reported good visual acuity in patients with low astigmatism treated with trifocal IOL [24].

Trifocal IOL have been associated with diffractive dysphotopsias, particularly under low-illumination conditions [25,26]. We evaluated our patients using the Refractive Cataract Surgery Survey (RCSS), a validated 10-question survey that evaluates key aspects of patient satisfaction and IOL performance, including dysphotopsias [27]. For the high myopia and low-to-moderate myopia groups, regardless of preoperative astigmatism, patients reported high levels of satisfaction with factors such as their quality of vision, level of glasses independence, and choice of lenses (Figure 5A). Regarding dysphotopsias, less than 50% (high myopia: n=6 eyes, low myopia: n=11 eyes) of patients reported glare or halos at night, and less than 10% (high myopia: n=6 eyes, low myopia: n=11 eyes) reported limitation of activities with glare and halos in both groups (Figure 5B). In patients with astigmatism in the low-to-moderate and high myopia groups, survey results followed a similar pattern of high patient satisfaction (Figure 5C). However, there was an increase in reported dysphotopsias in both groups and increased activity limitations from dysphotopsias in the low myopia group (Figure 5D). Overall, while both groups reported a similar burden of dysphotopsias, patients with low-to-moderate myopia tended to report limited activity from them more often, although not significant. Our survey findings are consistent with those of Espaillat et al., who suggest that patients with better baseline visual acuity have differing expectations compared to patients with worse baseline vision, who experience a more pronounced change in visual acuity after surgery [25]. 

Our study adds to the literature that with appropriate patient selection and modern surgical techniques, trifocal IOL can be safely and effectively used in highly myopic eyes with or without astigmatism. This is relevant, especially given the large global prevalence of high myopia and its projected increase in the near future. A major strength of our study is its real-world design, with all procedures performed by a single experienced surgeon using a standardized surgical platform and IOL model. This consistency reduces procedural variability, enhancing the reliability of the visual and refractive outcomes we observed. Additionally, we employed comprehensive outcome measures that included both objective refraction and visual acuity endpoints (UDVA, UNVA, SE) as well as a validated subjective patient satisfaction survey that covered key aspects of the patients' experience. Moreover, including patients with and without astigmatism in both myopia groups allowed for more nuanced subgroup comparisons. Similarly, including both low-to-moderate and high myopia patients allowed us to directly assess the correlation of clinical outcomes and the anatomical differences between the two groups.

Our findings in this study were limited by a relatively small sample size and the retrospective design. Additionally, we included only one type of trifocal IOL to provide consistency across patients. Thus, our findings are not generalizable to other multifocal or extended depth-of-focus (EDOF) lenses. Future studies should compare different classes of IOL (such as EDOF) as well as various other trifocal IOL technology in myopic eyes to help determine lens fitness for patients. Additionally, the occurrence of dysphotopsias may change in our cohort over time due to visual neuroadaptations. Future studies will aim to track patients over a longer follow-up period as well as a prospective design to generate more accurate data.

## Conclusions

Trifocal IOL may provide good outcomes in visual acuity and patient satisfaction in patients with high myopia, with or without astigmatism. Overall, visual outcomes and patient adaptations were excellent, even in the difficult-to-treat high astigmatism and high myopia group. Careful patient selection, modern surgical techniques, and accurate biometric targeting allowed for excellent postoperative vision and high satisfaction. Our findings support the applicability of trifocal IOL in patients with myopia. We also highlight the need to continue exploring methods to reduce the occurrence of dysphotopsias, improve surgical precision, and manage patient expectations, especially in eyes with increased anatomical complexity. Further studies are needed to validate our data in a larger cohort along with a wider array of presbyopia correcting lenses.
